# The Influence of Circulating Immune Cell and CA125 Dynamics on Neoadjuvant Therapy Selection for Advanced Ovarian Cancer

**DOI:** 10.3390/medicina60081290

**Published:** 2024-08-09

**Authors:** Alexandra Lazar, Ana Maria Popa, Cristina Orlov-Slavu, Horia-Teodor Cotan, Cristian Ion Iaciu, Cristina Mihaela Olaru, Oliver Daniel Schreiner, Romeo Cristian Ciobanu, Cornelia Nitipir

**Affiliations:** 1 Department 8—Clinical Medicine, Carol Davila University of Medicine and Pharmacy, 8 Sanitary Heroes Boulevard, 050474 Bucharest, Romania; alexandra.lazar@rez.umfcd.ro (A.L.); amy.popa@yahoo.com (A.M.P.); cristina.orlov-slavu@rez.umfcd.ro (C.O.-S.); horia-teodor.cotan@rez.umfcd.ro (H.-T.C.); cristian-ion.iaciu@rez.umfcd.ro (C.I.I.); miha0611@yahoo.com (C.M.O.); nitipir2003@yahoo.com (C.N.); 2 Department of Medical Oncology, Elias Emergency University Hospital, 17 Bd. Marasti, 011461 Bucharest, Romania; 3 Regional Institute of Oncology Iasi, 2-4 General Henri Mathias Berthelot Street, 700483 Iasi, Romania; oliver090598@yahoo.com; 4 Department 3—Medical Sciences, Grigore T. Popa University of Medicine and Pharmacy, 16 University Street, 700115 Iasi, Romania; 5 Department of Electrical Measurements and Materials, Gheorghe Asachi Technical University, 700050 Iasi, Romania

**Keywords:** circulating immune cell, ovarian cancer, neoadjuvant therapy, baseline NLR, post-therapeutic NLR

## Abstract

*Background and Objectives:* Ovarian cancer, including tubal and peritoneal cancer, is the third most common gynecological cancer and the leading cause of death from gynecological malignancies in developed countries. This study explores the prognostic value of the neutrophil-to-lymphocyte ratio (NLR) in determining the optimal duration of neoadjuvant chemotherapy (NACT) for advanced ovarian cancer. It also investigates the correlation between NLR dynamics and the KELIM score, a chemosensitivity marker, to enhance individualized therapeutic strategies. *Materials and Methods:* A total of 79 patients underwent NACT followed by interval debulking surgery (IDS) or palliative care. The data collected included demographic information, tumor characteristics, treatment modalities, and laboratory parameters. The baseline NLR (NLR-T0) and post-therapeutic NLR (NLR-T1) were calculated, and their variation (NLR∆) was analyzed. The KELIM score was determined using CA-125 values. *Results:* Patients with a high baseline NLR (≥2.5) had significantly worse progression-free survival (PFS) and overall survival (OS) compared to those with a low NLR (<2.5). A negative NLR∆ was associated with poorer PFS and OS. The KELIM score indicated a more effective treatment response, with higher scores correlating with better outcomes. The majority of patients achieved R0 resection, and those with favorable KELIM scores showed improved survival rates. *Conclusions:* The NLR is a valuable prognostic marker for assessing treatment response and guiding NACT duration in advanced ovarian cancer.

## 1. Introduction

Ovarian cancer, which encompasses tubal and peritoneal cancer within its classification, ranks as the third most prevalent gynecological cancer, following cervical and endometrial cancer [1]. It stands as the primary cause of death from gynecological cancer in developed nations [2,3]. Carcinomas, accounting for over 90% of malignant ovarian tumors, are believed to stem from the ovarian surface epithelium or the fallopian tube [4,5,6]. The five-year survival rate hovers around 49%, although patients with early-stage disease and specific histological subtypes tend to experience longer survival [7,8,9,10]. Around half of patients are diagnosed with distant or unresectable disease (FIGO stage IIIC-IV) initially; nonetheless, less common subtypes like clear cell and endometrioid cancer are more frequently identified at earlier stages [7,8,9,11]. Currently, the standard primary treatment for patients with advanced ovarian carcinoma is primary cytoreductive/debulking surgery (PDS), aiming to eliminate all visible tumor tissue, followed by platinum-based chemotherapy. Observational studies indicate that achieving optimal debulking with residual disease less than 1 cm is linked with improved overall survival [12]. Although the phase III CHORUS trial [13] revealed that overall survival (OS) following neoadjuvant chemotherapy (NACT) and interval debulking surgery (IDS) was comparable to that of patients undergoing primary surgery followed by adjuvant chemotherapy, recent data suggest that NACT is linked with a higher rate of achieving R0 resection and a reduced occurrence of postoperative comorbidities [14].

In recent years, there has been extensive research into the role of the tumor microenvironment (TME) in the development of carcinogenesis and metastases. Specifically, in ovarian cancer, tumor-promoting agents such as certain T lymphocytes and tumor-associated macrophages found in ascites fluid have been shown to facilitate infiltration and metastasis. Notably, one of the primary contributors to disease progression and treatment resistance is the suppression of the immune response. This immune response, mediated through the TME, involves CD4+ T cells, CD8+ T cells, and NK cells, which are directly hindered not only by tumor cells but also by immunosuppressive regulatory T cells (Tregs), immature dendritic cells (DCs), tumor-associated macrophages (TAMs), and myeloid-derived suppressor cells (MDSCs).

A defining aspect of the tumor microenvironment (TME) in ovarian cancer is the involvement of resident host cells, particularly activated mesothelial cells lining the peritoneal cavity in abundance, alongside adipocytes of the omentum, which serve as the preferred site for metastatic lesions. Another critical element is the peritoneal fluid, facilitating the transcoelomic spread of tumor cells to other pelvic and peritoneal organs. This ascites contains a wealth of tumor-promoting soluble factors, extracellular vesicles, and detached cancer cells, as well as significant populations of T cells, tumor-associated macrophages (TAMs), and other host cells, all collaborating with resident host cells to bolster tumor progression and evade immune surveillance [15]. The neutrophil-to-lymphocyte ratio (NLR) is an inflammatory marker that has been extensively researched as a prognostic factor in cancer [16,17]. A meta-analysis [18] including 2919 patients has reported that the values of inflammatory markers of the NLR were associated with ovarian cancer survival. Therefore, inflammatory markers can potentially serve as prognostic biomarkers. Moreover, a decrease in the NLR has been suggested as a predictive factor for neoadjuvant chemotherapy response in advanced ovarian cancer [19].

Biomarkers identify circulating tumor elements, proteins overexpressed by tumors, or components of the immune response to the tumor in body fluids such as blood and urine. Currently, protein biomarkers for ovarian cancer are used to monitor disease progression [20]. The role of CA125 in predicting suboptimal cytoreduction was investigated in a prospective multicenter trial involving stage III and IV patients undergoing primary cytoreductive surgery. Six criteria, including CA125 levels above 500 U/mL, were associated with suboptimal cytoreduction (residual cancer > 1 cm) [21]. More recently, the KELIM (rate of elimination of CA-125) score has been used to evaluate changes in serum CA125, potentially indicating treatment efficacy. Mathematical modeling allows for the calculation of equations describing the longitudinal changes in tumor biomarkers. This model-based population kinetic approach is particularly relevant as it enables the determination of individual kinetic profile parameters from a few time points, minimizing the impact of inter- and intra-individual variability in time points and assays. The KELIM score was validated in the GOG-218 study [22] as well as several retrospective studies [23,24].

This study aims to explore the significance of changes in the neutrophil-to-lymphocyte ratio (NLR) in determining the optimal duration of neoadjuvant chemotherapy (NACT) for patients diagnosed with ovarian cancer. Recognizing the NLR as a potential prognostic marker in various cancers, its precise relevance in guiding NACT duration in ovarian cancer remains uncertain. By investigating the relationship between fluctuations in NLR levels and treatment response, this study seeks to determine if NLR dynamics can reliably indicate the appropriate duration of NACT regimens. Additionally, the present study aims to establish if there is a correlation between the NLR as an inflammatory biomarker and the KELIM score as a chemosensitivity marker. The findings from this study hold promise for enhancing treatment precision and patient outcomes by facilitating individualized therapeutic strategies in the management of ovarian cancer.

## 2. Materials and Methods

### 2.1. Studied Population

All patients involved in this retrospective study received treatment and follow-up at the Oncology Department of Elias Emergency University Hospital in Bucharest, Romania, from January 2016 to January 2024. All participants in this study were diagnosed with stage III-IV ovarian cancer. Treatment for these patients involved neoadjuvant chemotherapy with carboplatin (AUC = 6, the area under the curve is 6) and paclitaxel 175 mg/m^2^ every 3 weeks for 3–6 cycles, followed by either interval debulking surgery (IDS) or palliative care in the rare cases of non-chemotherapy-responders. Patients were also treated with adjuvant chemotherapy after IDS. Adjuvant chemotherapy consisted of carboplatin (AUC = 6, the area under the curve is 6) and paclitaxel 175 mg/m^2^ every 3 weeks. The inclusion criteria required a confirmed positive diagnosis of epithelial ovarian cancer (EOC) through histopathological and immunohistochemical assessments, along with precise clinical staging (stage III–IV). Pathological staging was conducted by an experienced pathologist utilizing the American Joint Committee on Cancer (AJCC) TNM Staging Classification for Ovarian, Fallopian Tube, and Primary Peritoneal Cancer 8th edition, 2017.

**Figure 1:**
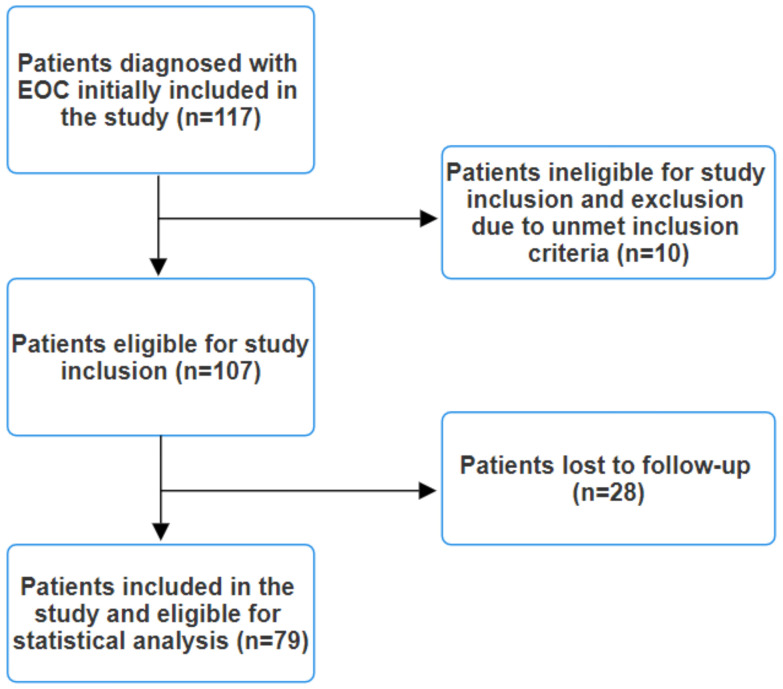
Study flow diagram.

Clinical staging involved comprehensive CT or MRI scans of the chest, abdomen, and pelvis, along with brain MRI scans for symptomatic patients. Lesions suspected to be metastases but not definitively identified as such on conventional imaging underwent biopsy for histopathological and immunohistochemical confirmation or an 18F-FDG PET/CT scan was performed. The exclusion criteria encompassed the presence of any indicators or symptoms suggestive of infection, such as elevated procalcitonin levels, leukocytosis, fever, malaise, abnormal chest radiography, or positive cultures from blood, urine, or pharyngeal exudate, as these factors could potentially affect the final results. Patients with an immunocompromised status or autoimmune pathologies were also excluded. Additionally, individuals undergoing corticosteroid therapy were excluded from this study. Patients diagnosed with other synchronous cancers were similarly excluded. Initially, 117 patients with EOC were included in this study. However, after applying the exclusion criteria, a total of 79 patients remained eligible (Figure 1).

### 2.2. Data Collection

We conducted retrospective data collection encompassing demographic information (age, sex, family history), tumor characteristics (histology, TNM stage, molecular biomarkers such as BRCA1/2 and homologous repair deficiency), treatment modalities, and laboratory parameters (complete blood count, liver function tests, carbohydrate antigen 125 (CA125)).

We defined the baseline neutrophil-to-lymphocyte ratio (noted as NLR-T0) as the ratio derived from complete blood counts obtained within three days before initiating neoadjuvant therapy. The post-therapeutic NLR (noted as NLR-T1) was designated as the NLR obtained after the completion of neoadjuvant therapy. We also measured the variation in the NLR between these two time points, noted as NLR∆ (NLR-T0 minus NLR-T1). A stable or positive NLR∆ indicated that the NLR remained unchanged or decreased, while a negative NLR∆ indicated an increase in the NLR between the baseline and post-therapeutic NLR. The calculation of the NLR involved dividing the absolute neutrophil count by the absolute lymphocyte count. The absolute neutrophil count (ANC) was determined by multiplying the total white blood cell count by the percentage of neutrophils and dividing by 100. Conversely, the absolute lymphocyte count was computed by multiplying the total white blood cell count by the percentage of lymphocytes among white blood cells.

The KELIM score was calculated using at least three CA-125 values within the first 100 days of neoadjuvant chemotherapy, notably at baseline and pre-cycle 2 and 3, using a validated calculation formula available in [25]. A KELIM score of <1 was considered unfavorable, with lower chemosensitivity, while a KELIM score of ≥1 was considered favorable. The KELIM score varies between 0.25 and 2.5 points.

### 2.3. Statistical Analysis

Statistical analyses were conducted using SPSS software version 26.0. Descriptive statistics were employed to report patient and disease characteristics. Differences between groups were assessed using Pearson’s Chi-squared test or Fisher’s exact test for categorical variables, while the two-sample T-test or Wilcoxon rank-sum test was utilized for continuous variables. Median survival time was evaluated using the Kaplan–Meier method, and survival distributions between groups were compared using the log-rank test to identify significant differences in survival outcomes. Univariate and multivariate Cox regression models were employed to explore the relationship between elevated levels of the NLR and the risk of death or recurrence among patients with epithelial ovarian cancer (EOC), allowing for adjustments for potential confounding variables. Overall survival was calculated from the time of diagnosis to the date of death, while progression-free survival was calculated from treatment initiation until the first documented progression according to RECIST criteria. The overall response rate (ORR) was defined as the proportion of patients achieving partial or complete response to the first line of therapy, with partial response, complete response, and stable disease categorized as favorable responses, and progressive disease considered unfavorable. Continuous variables with a normal distribution were expressed as mean and standard deviation (SD), while those without a normal distribution were presented as median and quartiles. Categorical variables were expressed as counts (*n*) and percentages (%). Results with a *p*-value less than 0.05 were considered statistically significant.

## 3. Results

### 3.1. Patients’ Characteristics

Out of 117 cases of epithelial ovarian cancer (EOC) patients treated with systemic therapy, 79 were included in this analysis after applying the inclusion/exclusion criteria. The average age of these patients was 63.7 years (SD = 7.52), ranging from 47 to 77 years. Most patients had either locally advanced unresectable disease (stage IIIC; *n* = 34, 43%) or metastatic disease (stage IV; *n* = 27, 34.2%) at diagnosis. Additionally, there were 8 patients (10.1%) with stage IIIA disease and 10 patients (12.7%) with stage IIIB disease. Among the stage IV patients, 12 (44.4%) had oligometastatic disease (1–5 metastatic sites) and 15 (55.6%) had disseminated metastatic disease.

A total of 63 patients (79.7%) underwent interval debulking surgery (IDS), while 16 patients (20.3%) were not eligible for cytoreductive surgery even after neoadjuvant chemotherapy (NACT). Resection rates varied, with 51 patients (81%) achieving R0 resections, indicating complete tumor removal, and 12 patients (15.2%) achieving R1 resection, indicating microscopic residual disease.

Bevacizumab therapy was rarely used in the neoadjuvant setting, with only 12 patients (15.2%) receiving it, while the remaining 67 patients (84.8%) did not. BRCA1 and BRCA2 mutations were detected in 18 patients (22.8%), with the remaining 61 patients (77.2%) showing no BRCA mutations. Additionally, 13 patients (16.5%) were homologous recombination deficient (HRD), while the other 66 patients (83.5%) were not.

The number of neoadjuvant cycles varied: 30 patients (38%) received six cycles of carboplatin and paclitaxel, 26 patients (32.9%) received three cycles, 6 patients (7.6%) received four cycles, and 1 patient (1.3%) received five cycles. The remaining 16 patients (20.3%) received six cycles of chemotherapy either with bevacizumab followed by maintenance therapy with bevacizumab, bevacizumab and PARP inhibitors, or just PARP inhibitors, depending on BRCA1/2 and HR status. Performance status was also analyzed, with 15 patients (19%) having an ECOG score of ≥2, 43 patients (54.4%) having an ECOG score of 1, and 21 patients (26.5%) having a score of 0. In this cohort, the KELIM score was calculated for all patients to assess treatment response. The distribution of the KELIM scores is as follows: 64 patients (81%) had a KELIM score ≥ 1 and 15 patients (19%) had a KELIM score < 1. These data indicate that the majority of the patients exhibited a KELIM score ≥ 1, suggesting a more effective treatment response in terms of CA-125 elimination. The OS for the entire cohort was 47 months (95% CI 43.660–50.340), while the PFS time was 19 months (95% CI 17.626–20.374). These clinical characteristics and molecular parameters at baseline are detailed in Table 1.

### 3.2. Clinical Outcome According to Baseline NLR-ROC Analysis

**Figure 2:**
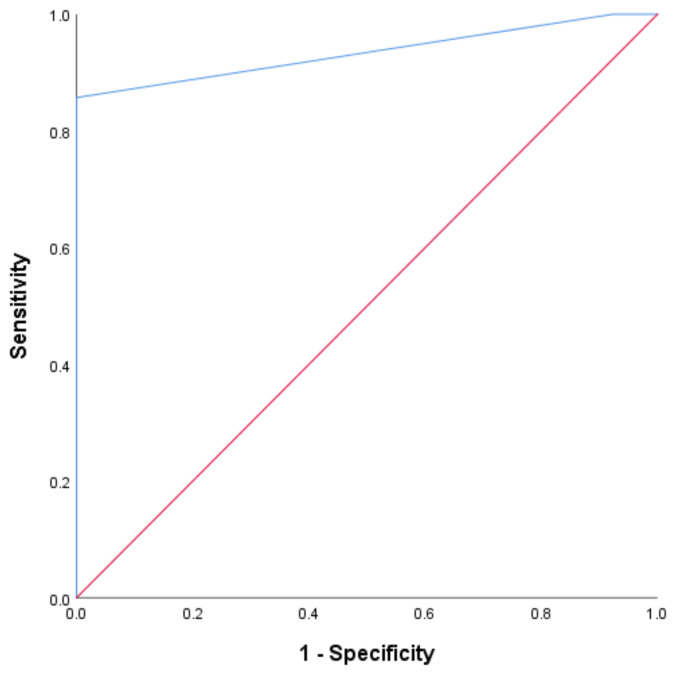
The ROC curve for the baseline NLR. The plot of the receiver operating characteristic curve of the NLR assessed at baseline as a predictor of objective tumor response.

ROC analysis was conducted to determine the optimal cutoff value for the NLR as a predictor of objective tumor response. The ROC curve for the baseline NLR demonstrated high diagnostic accuracy, with an AUC of 0.900 (95% CI 0.832 to 0.967, *p* < 0.0001). The Youden index identified the best cutoff at 2.5, with a sensitivity of 100% and a specificity of 92% (Figure 2).

**Figure 3:**
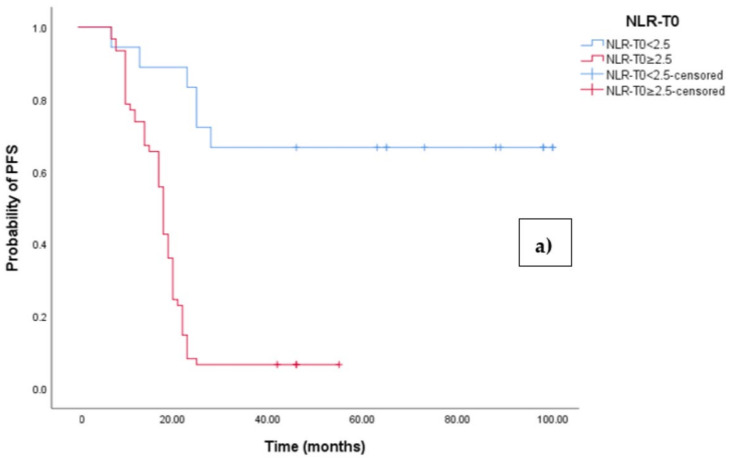

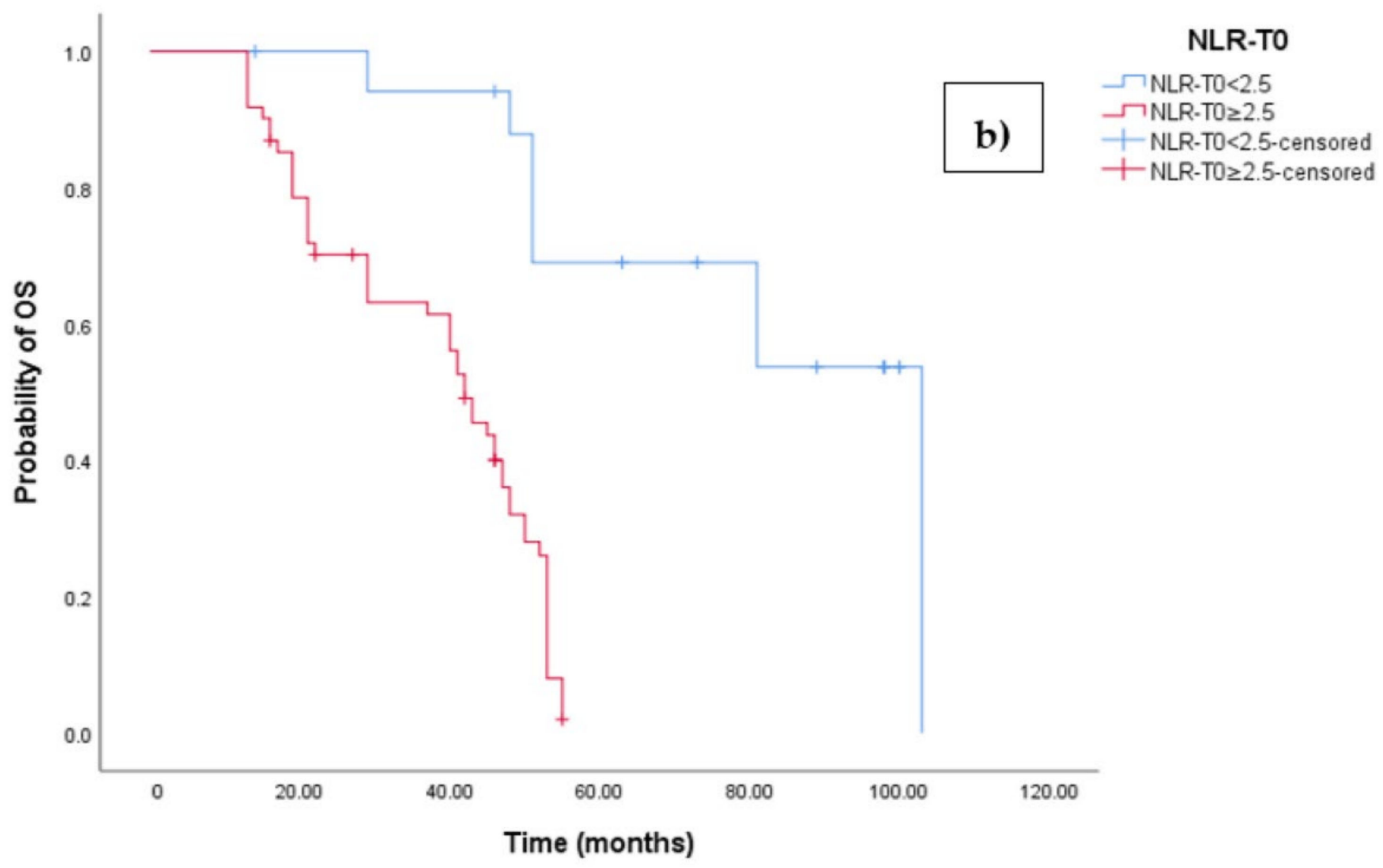
(**a**) Comparative analysis of Kaplan–Meier curves between Low NLRT0 and high NLRT0 patients for progression-free survival (PFS). (**b**) Comparative analysis of Kaplan–Meier curves between Low NLRT0 and high NLRT0 patients for overall survival (OS).

The Kaplan–Meier survival analysis revealed that EOC patients with a high baseline NLR (NLR-T0 ≥ 2.5) exhibited significantly worse progression-free survival (PFS), with a median PFS time of 18 months (95% CI 17.054–18.946, *p* < 0.0001; see Figure 3a). In contrast, those with a low baseline NLR (NLR-T0 < 2.5) did not reach the median PFS time. Similarly, the survival analysis indicated that EOC patients with a high NLR-T0 experienced a shorter overall survival (OS) time of 42 months (95% CI 36.856–47.144, *p* < 0.0001; see Figure 3b) compared to those with a low NLR-T0, who exhibited a median OS time of 103 months (95%CI 88.523–110.227).

### 3.3. Clinical Outcome According to NLRΔ

**Figure 4:**
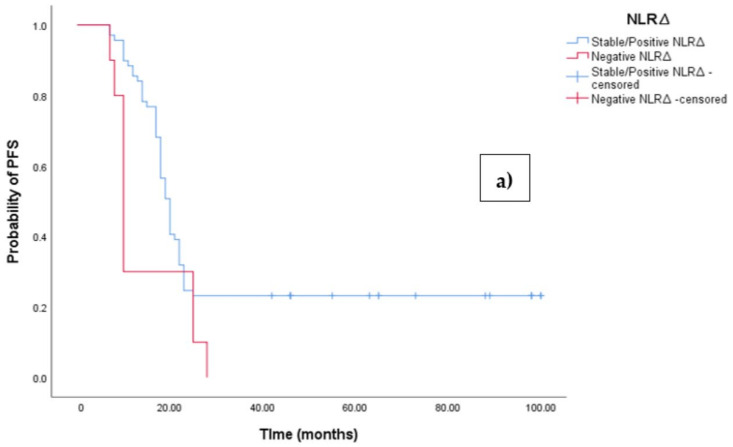

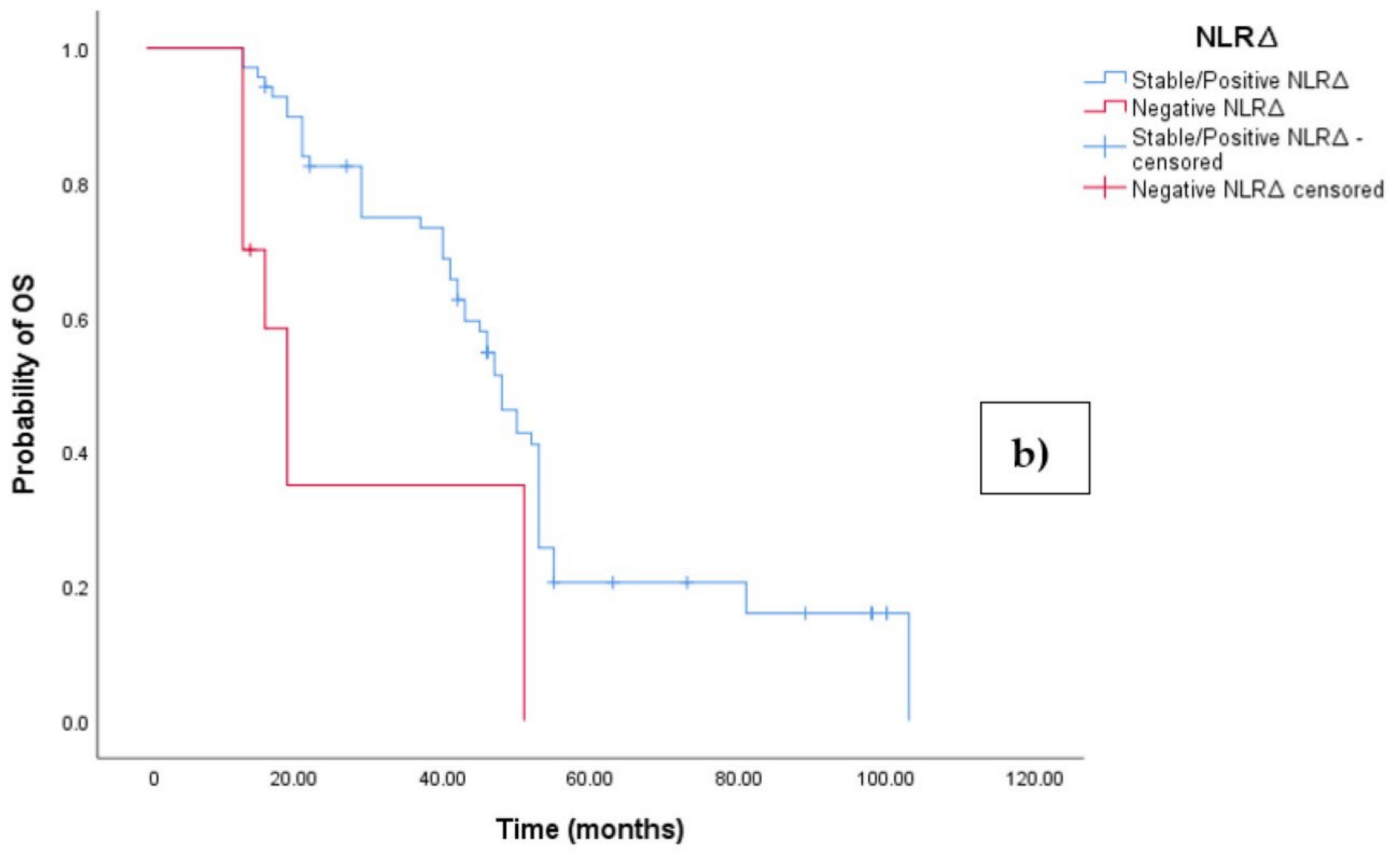
(**a**) Comparative analysis of Kaplan–Meier curves between negative NLRΔ and positive/stable NLRΔ patients for progression-free survival (PFS). (**b**) Comparative analysis of Kaplan–Meier curves between negative NLRΔ and positive/stable NLRΔ patients for overall survival (OS).

The Kaplan–Meier survival analysis revealed that EOC patients with a negative NLR∆ exhibited significantly worse progression-free survival (PFS), with a median PFS time of 10 months (95%CI 8.864–11.136, *p* = 0.027; see Figure 4a). In contrast, those with a positive/stable NLR∆ had a median PFS time of 20 months (95%CI 18.546–21.454, *p* = 0.027; see Figure 4a). Similarly, the survival analysis indicated that EOC patients with a negative NLR∆ experienced a shorter overall survival (OS) time of 19 months (95% CI 14.963–23.037, *p* = 0.002; see Figure 4b) compared to those with a positive/stable NLR∆, who exhibited a median OS time of 48 months (95%CI 43.940–52.060, *p* = 0.002).

### 3.4. Clinical Outcome According to KELIM Score

**Figure 5:**
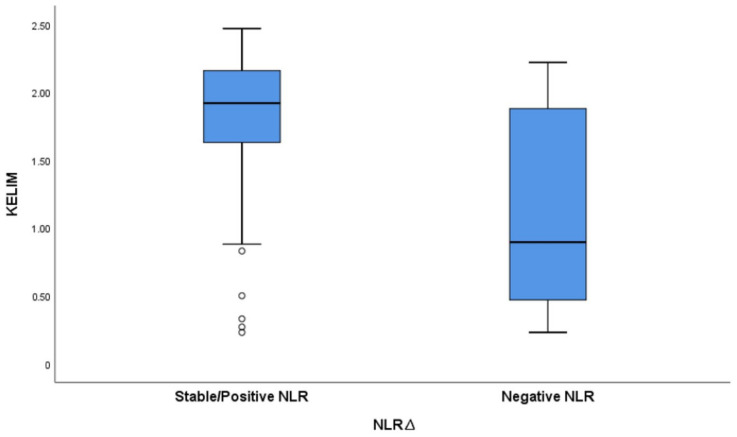
Boxplot graph exhibiting variation in KELIM score based on NLRΔ.

We have calculated the KELIM score using CA125 measured at baseline before NACT initiation, and before cycle 2 and 3. The mean KELIM value was 1.3 (SD = 0.35, range 0.27–2.43). The KELIM score varied between the positive/stable NLR∆ group and the negative NLR∆ group, with a mean KELIM score of 1.9 and 0.67, respectively (Figure 5).

**Figure 6:**
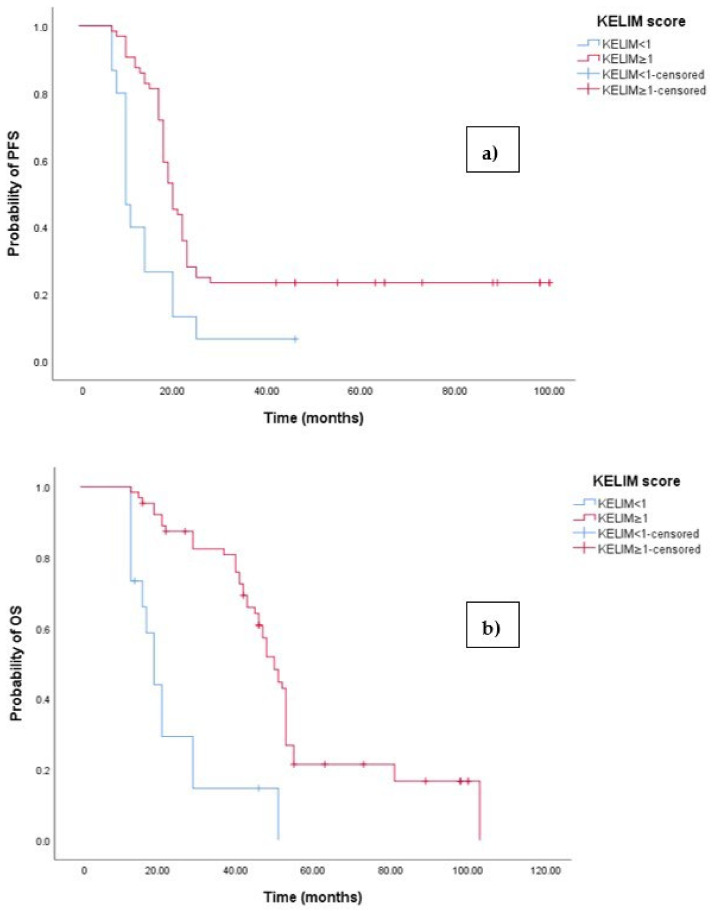
(**a**) Comparative analysis of Kaplan–Meier curves between patients with KELIM score ≥ 1 and KELIM score < 1 for progression-free survival (PFS). (**b**) Comparative analysis of Kaplan–Meier curves between patients with KELIM score ≥ 1 and KELIM score < 1 for overall survival (OS).

The Kaplan–Meier survival analysis revealed that EOC patients with a KELIM score ≥ 1 exhibited significantly better progression-free survival (PFS), with a median PFS time of 23 months (95% 17.658–22.342, *p* < 0.001; see Figure 6a). In contrast, those with a KELIM score < 1 had a PFS time of 11 months (95% CI 8.106–12.894, *p* < 0.001; see Figure 6a). Similarly, the survival analysis indicated that EOC patients with a KELIM score < 1 experienced a shorter overall survival (OS) time of 20 months (95% CI 15.856–47.144, *p* < 0.0001; see Figure 3b) compared to those with a low NLR-T0, who exhibited a median OS time of 103 months (95%CI 88.523–110.227).

### 3.5. Multivariate Analysis to Identify Predictors for Increased Risk of Death and Tumor Progression

The prognostic value of the KELIM score and NLR∆ have been confirmed in the studied population through multivariate analysis (Cox regression). The hazard ratio (HR) for death among patients with a negative NLR∆ was 3.750 (95% CI 2.220–6.421, *p* < 0.001; Table 2), while the HR for cancer progression was 3.177 (95% CI 2.225–7.671, *p* < 0.001; Table 2). Similarly, patients with a KELIM score of less than 1 had an increased risk of both death, with an HR of 1.910 (0.852–4.282), and cancer progression, with an HR of 2.185 (1.865–6.722). Although the baseline NLR demonstrated prognostic value in the univariate Kaplan–Meier analysis, it did not retain prognostic significance in the multivariate analysis, with an HR of 2.173 (1.810–2.610, *p* = 0.454) for OS and an HR of 1.927 (1.602–2.319, *p* = 0.321) for PFS. Other significant variables associated with a worse OS and PFS are BRCA mutational status and resection rate.

We have also examined the overall response rate (ORR) categorized by the KELIM score and NLRΔ. We have defined a favorable outcome as a partial response, a complete response, or stable disease, and an unfavorable outcome as progressive disease. The majority of positive/stable NLRΔ patients (*n* = 68, 98.5%, see Table 3) showed a favorable response, whereas a smaller percentage of patients in the negative NLRΔ group (*n* = 4, 40%, see Table 3) exhibited a favorable response. A total of 64 patients (100%) with a KELIM score ≥ 1 showed a favorable response, while only 8 (53.3%) patients with a KELIM score < 1 showed a favorable response.

### 3.6. Selection of Maintenance Therapy

**Figure 7:**
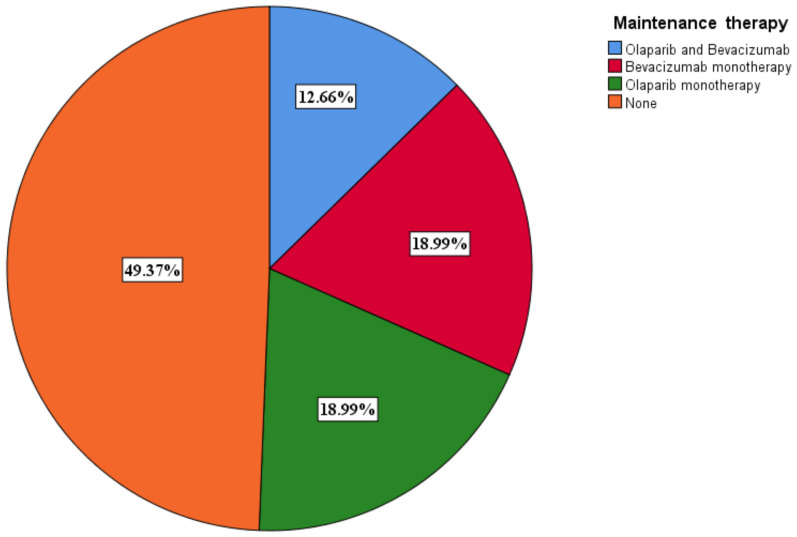
Maintenance therapy distribution among patients.

A total of 40 (50.6%) patients benefited from maintenance therapy, with 10 (12.7%) receiving olaparib and bevacizumab and 15 (19%) receiving olaparib (Figure 7). The PARPi only patients benefited from olaparib. Olaparib monotherapy was given to BRCA 1/2 mutant only patients. Olaparib was given at a dose of 600 mg a day, while bevacizumab was given at a dose of 15 mg/kg every 3 weeks.

We assessed the effect of maintenance therapy on OS and PFS across different subgroups. For patients in the KELIM < 1 group who had completed adjuvant therapy, the OS times were as follows: 13 months (95% CI 9.631–25.225) for those receiving olaparib and bevacizumab, 26 months (95% CI 10.970–41.030) for those on bevacizumab monotherapy, and 11 months (95% CI 7.397–16.634. *p* = 0.002) for those on olaparib monotherapy. In contrast, patients in the KELIM ≥ 1 group had the following OS times: 45 months (95% CI 39.456–50.544, *p* = 0.002) for those receiving olaparib and bevacizumab, 21 months (95% CI 12.444–32.236, *p* = 0.002) for those on bevacizumab monotherapy, and 51 months (95% CI 46.143–55.857) for those on olaparib monotherapy.

PFS mirrored the trends observed in OS, with bevacizumab showing superior outcomes in patients with a KELIM score < 1. The observed PFS times were as follows: 10 months (95% CI 5.441–15.231) for patients receiving olaparib and bevacizumab, 17 months (95% CI 11.785–24.196) for those on bevacizumab monotherapy, and 14 months (95% CI 9.737, *p* = 0.006) for those on olaparib monotherapy. In contrast, patients in the KELIM ≥ 1 group had the following PFS times: 18 months (95% CI 11.070–24.930, *p* = 0.006) for those receiving olaparib and bevacizumab, 16 months (95% CI 13.686–20.314, *p* = 0.006) for those on bevacizumab monotherapy, and 25 months (95% CI 20.772–29.228) for those on olaparib monotherapy.

## 4. Discussion

Inflammatory processes can pave the way for a microenvironment conducive to tumor growth in ovarian cancer, evolving into a widespread condition in later stages [26]. Symptoms commonly seen in advanced ovarian cancer patients are often attributed to this systemic inflammation, which significantly influences patient outcomes. These symptoms usually include declines in nutrition, functionality, and immune response [26]. Over recent decades, numerous researchers have delved into various markers of systemic inflammation, exploring their potential in categorizing cancer patients based on disease stage [27]. Consequently, parameters related to inflammation are increasingly gaining traction as promising tools for prognosticating cancer outcomes [28].

Neoadjuvant chemotherapy (NACT) has emerged as a valuable treatment strategy for patients with advanced ovarian cancer, offering the potential for tumor debulking and improved surgical outcomes [29]. In this study, we investigated the role of the neutrophil-to-lymphocyte ratio (NLR) as a predictive biomarker in the context of NACT for ovarian cancer.

Our findings reveal a significant association between NLR dynamics and treatment response. Notably, we observed that a decreasing NLR from baseline to the end of NACT is strongly correlated with favorable outcomes. This observation underscores the potential utility of the NLR as a prognostic indicator in ovarian cancer management, particularly in the neoadjuvant setting.

The inverse relationship between NLR reduction and treatment response aligns with previous studies [19], highlighting the role of systemic inflammation in cancer progression and treatment resistance. An elevated NLR reflects a state of heightened inflammation, which has been implicated in tumor promotion and the evasion of immune surveillance. Thus, a decrease in the NLR following NACT may signify a reduction in the pro-tumoral inflammatory microenvironment, potentially enhancing treatment efficacy and patient outcomes.

High-grade serous ovarian cancer typically exhibits high sensitivity to chemotherapy; however, a small subset of patients, estimated at less than 10%, show refractoriness to first-line therapy, suggesting the presence of inherent resistance mechanisms. Despite achieving clinical remission, the majority of patients eventually experience disease relapse, indicating a complex interplay of factors contributing to therapy failure [30]. While some individuals develop acquired chemoresistance, characterized by an insensitivity to previously effective treatments, a significant portion of patients experience recurrence under the same therapeutic regimen. This recurrence of lesions with similar chemosensitivity profiles as the primary tumor suggests a distinct mechanism of therapy failure, one that is distinct from intrinsic or acquired resistance. However, the precise mechanisms underlying this phenomenon of transient chemoresistance remain elusive.

The selection of therapy is currently of utmost importance in treating locally advanced ovarian cancer. In this study, we have shown that an increase in the NLR after neoadjuvant chemotherapy was associated with improved PFS and OS. Similar data were reported in a study of 72 ovarian cancer patients undergoing chemotherapy, where a high NLR was linked to worse OS and poor treatment response. The multivariate analysis showed that an elevated NLR is a weak predictor in this group [31].

Similarly, a larger retrospective study by Feng et al. [32] on 875 high-grade serous ovarian cancer patients found that high preoperative NLR levels were associated with advanced clinical stage, higher CA125 levels, extensive ascites, poor cytoreduction outcomes, and chemotherapy resistance. A univariate analysis showed that an elevated NLR negatively correlated with overall survival (OS) and progression-free survival (PFS), while in a multivariate analysis, a high NLR remained an independent prognostic factor for PFS but not OS. These findings are similar to our own study and suggest that the NLR may indicate tumor burden and clinical prognosis in HGSC patients and should be considered a prognostic marker.

The relation between the NLR and chemotherapy response has been rarely studied. In our study, we evaluated the association between the KLIM score and NLR. The KLIM score is a validated predictive score showing that a CA125 decrease from baseline was associated with an increased ORR as well as OS and PFS [33].

In this study, the majority of patients (81%) had a KELIM score greater than 1, which was linked to increased progression-free survival (PFS) and overall survival (OS), as well as a higher response rate. Interestingly, patients with a KELIM score > 1 who received bevacizumab maintenance monotherapy had worse outcomes compared to those with a KELIM score < 1. These results are consistent with the GOG-218 validation study [34], which indicated that bevacizumab is primarily beneficial for patients with poorly chemosensitive tumors. There was no observed benefit in patients with favorable KELIM scores. The greatest benefit from bevacizumab in terms of PFS and OS (with an absolute OS benefit of approximately 6 to 9 months) was seen in patients with high-risk diseases (stage IV or incompletely resected stage III) who also had an unfavorable KELIM score.

Our findings suggest that the KELIM score is an independent prognostic factor for OS and PFS, irrespective of the completeness of debulking surgery. A high KELIM score, indicating a rapid decline in CA-125 levels, is associated with a better response to chemotherapy and improved survival outcomes. This suggests that tumors which respond quickly to initial chemotherapy are more likely to be chemosensitive, leading to prolonged progression-free survival (PFS) and overall survival (OS). Studies have shown that patients with high KELIM scores have a significantly higher median PFS and OS compared to those with low KELIM scores, underscoring its utility as a prognostic marker [35]. The KELIM score’s role as a prognostic factor for overall survival (OS) and progression-free survival (PFS) may be attributed to the fact that patients with a higher KELIM score, indicating chemosensitive ovarian cancer, demonstrated higher rates of complete interval debulking surgery (IDS). Conversely, a low KELIM score, indicating a slower decline in CA-125 levels, suggests a poorer response to chemotherapy and worse prognosis. This can prompt oncologists to consider alternative treatment strategies early in the treatment course. For instance, patients with low KELIM scores may benefit from more aggressive or novel therapeutic approaches, including targeted therapies or participation in clinical trials.

Elevated NLR values have been associated with poor prognosis in advanced ovarian cancer. A high NLR indicates a higher neutrophil count relative to lymphocytes, suggesting a state of systemic inflammation, which can promote tumor progression and suppress anti-tumor immune responses. Inflammatory cells, particularly neutrophils, can enhance tumor growth, angiogenesis, and metastasis through the release of pro-inflammatory cytokines and other factors.

Studies have demonstrated that a high pre-treatment NLR is correlated with decreased overall survival (OS) and progression-free survival (PFS) in patients with advanced ovarian cancer [36]. For instance, an analysis [28] of ovarian cancer patients treated with chemotherapy found that those with a high NLR had significantly shorter median OS and PFS compared to those with a low NLR. This relationship remains significant even after adjusting for other known prognostic factors, suggesting that the NLR is an independent prognostic marker. Moreover, Cho et al. [28] reported that the preoperative NLR, when combined with CA125, may serve as a simple and cost-effective method for identifying ovarian cancers. This aligns with our own hypothesis.

While both the NLR and KELIM score were associated with survival in advanced ovarian cancer, no significant correlation was seen between the two variables. This lack of correlation is likely because the NLR and NLR∆ (as defined above) are associated with tumor burden and the extent of the cancer-induced immune response and not with the ORR to chemotherapy. This is suggested by the fact that 40% of NLR∆ negative patients had a favorable response.

While there are few studies reporting the role of the NLR and KELIM score in the context of neoadjuvant therapy in ovarian cancer, there are several limitations to our study. This retrospective study on ovarian cancer, examining the role of the KELIM score and NLR in the context of neoadjuvant therapy, has several limitations. First, there is an inherent selection bias due to the reliance on pre-existing medical records, which might not represent the broader ovarian cancer population and potentially skew the results. The quality and completeness of recorded data can vary, leading to inaccuracies, and missing or incomplete data can affect the reliability of our findings. Additionally, retrospective analyses are often limited in their ability to account for all confounding variables, meaning unmeasured factors might influence the observed associations between the KELIM score, the NLR, and treatment outcomes. The temporal sequence of events can also be ambiguous, making it challenging to determine whether changes in the NLR and KELIM score are causes or consequences of treatment outcomes.

Furthermore, results from a single institution or specific patient population may not be generalizable to other settings, as variations in treatment protocols and patient demographics can limit the applicability of our findings to broader populations. The present study might have limited statistical power due to the small sample size or heterogeneity within the cohort, affecting the detection of significant associations. Observer bias is another concern, where researchers’ expectations or knowledge about patient outcomes might influence data collection or interpretation. Variations in neoadjuvant therapy regimens, surgical techniques, and postoperative care among patients introduce heterogeneity, complicating the analysis and interpretation of the results. The absence of random assignment means that compared groups may differ in ways beyond the variables of interest, potentially confounding the results. These limitations highlight the need for cautious interpretation of our findings and underscore the importance of future prospective studies with robust designs to validate these associations and provide more definitive insights into the prognostic and predictive roles of the KELIM score and NLR in ovarian cancer treated with neoadjuvant therapy.

## 5. Conclusions

This study explores the prognostic value of the neutrophil-to-lymphocyte ratio (NLR) in determining the optimal duration of neoadjuvant chemotherapy (NACT) for advanced ovarian cancer. It also investigates the correlation between NLR dynamics and the KELIM score, a chemosensitivity marker, to enhance individualized therapeutic strategies. A total of 79 patients underwent NACT followed by interval debulking surgery (IDS) or palliative care. The data collected included demographic information, tumor characteristics, treatment modalities, and laboratory parameters. The baseline NLR (NLR-T0) and post-therapeutic NLR (NLR-T1) were calculated, and their variation (NLR∆) was analyzed. The KELIM score was determined using CA-125 values.

It was noticed that the patients with a high baseline NLR (≥2.5) had significantly worse progression-free survival (PFS) and overall survival (OS) compared to those with a low NLR (<2.5). A negative NLR∆ was associated with poorer PFS and OS. The KELIM score indicated a more effective treatment response, with higher scores correlating with better outcomes. The majority of patients achieved R0 resection, and those with favorable KELIM scores showed improved survival rates.

The NLR is a valuable prognostic marker for assessing treatment response and guiding NACT duration in advanced ovarian cancer. The combination of NLR dynamics and the KELIM score can enhance treatment precision, facilitating individualized therapeutic approaches and improving patient outcomes. Further prospective studies are warranted to validate these findings.

## Figures and Tables

**Table 1:** Clinical characteristics and molecular parameters at baseline.

Characteristics	Patients Diagnosed with EOC (*n* = 79)
Age, years, mean (SD)	65.4 (±5.43)
Clinical stage, *n* (%)	
Stage IIIA	10 (2.5%)
Stage IIIB	12 (6.3%)
Stage IIIC	34 (43%)
Stage IV	27 (34.2%)
ECOG, *n* (%)	
0	21 (26.5%)
1	43 (54.4%)
≥2	15 (19%)
KELIM score, *n* (%)	
KELIM ≥ 1	64 (81%)
KELIM < 1	15 (19%)
BRCA 1/2 mutational status, *n* (%)	
Wild-type	61 (77.2%)
Mutant	18 (22.8%)
Homologous recombination status, *n* (%)	
Homologous recombination deficient (HRD)	13 (16.5%)
Homologous recombination proficient (HRP)	66 (83.5%)
Resction status	
R0	51 (64.6%)
R1	12 (15.2%)
Unresectable	16 (20.3%)
Bevacizumab treatment	
Bevacizumab +	12 (15.2%)
Bevacizumab -	67 (84.8%)
Number of NACT cycles	
3 cycles	26 (32.9%)
4 cycles	6 (7.6%)
5 cycles	1 (1.3%)
6 cycles	30 (38%)
Baseline NLR	
≥2.5	18 (22.8%)
<2.5	61 (77.2%)
NLR∆	
Positive/Stationary	69 (87.3%)
Negative	10 (12.7%)
Median PFS (months)	19 months (95%CI 17.626–20.374)
Median OS (months)	47 months (95% CI 43.660–50.340)

ECOG = Eastern Cooperative Oncology Group, BRCA1/2 = Breast Cancer gene 1/2, R0 = complete resection, R1 = microscopic invaded margins, NLR = neutrophil-to-lymphocyte ratio, PFS = progression-free survival, and OS = overall survival.

**Table 2:** Multivariate Cox regression analyses to identify predictors for increased risk of death and tumor progression.

Test Variables	OS HR (95% CI)	*p*-Value	PFS HR (95% CI)	*p*-Value
NLR∆ positive/stable (ref.)/negative	3.750 (2.220–6.421)	<0.001	3.177 (2.225–7.671)	<0.001
BRCA1/2 mutational status mutant (ref.)/wild-type	1.352 (1.120–1.631)	0.003	1.478 (1.234–1.763)	0.001
HRD (ref.)/HRP	1.523 (1.293–1.842)	0.006	1.411 (1.192–1.682)	0.006
ECOG 0-1 (ref.)/2	2.124 (1.753–2.573)	0.072	1.589 (1.345–1.922)	0.143
KELIM score ≥ 1 (ref.)/KELIM score < 1	1.910 (0.852–4.282)	0.006	2.185 (1.865–6.722)	0.006
Stage III (ref.)/Stage IV	2.301 (1.904–2.792)	0.257	1.519 (1.284–1.830)	0.226
R0 resection (ref.)/R1 resection	2.456 (2.004–3.002)	<0.001	1.772 (1.504–2.223)	0.003
Cancer antigen 125 (CA 125) low (ref.)/high	1.392 (1.182–1.643)	0.186	1.576 (1.314–1.889)	0.247
Age < 65 years (ref.)/>65 years	1.382 (1.157–1.652)	0.935	1.198 (0.537–1.517)	0.653
No. of NACT: 3 cycles (ref.)/6 cycles	1.653 (1.389–1.967)	0.893	1.548 (1.293–1.856)	0.785
Baseline NLR low (ref.)/baseline NLR high	2.173 (1.810–2.610)	0.454	1.927 (1.602–2.319)	0.321

ECOG = Eastern Cooperative Oncology Group, BRCA1/2 = Breast Cancer gene 1/2, R0 = complete resection, R1 = microscopic invaded margin, NLR = neutrophil-to-lymphocyte ratio, HRD = homologous repair deficiency, HRP = homologous repair proficiency, PFS = progression-free survival, and OS = overall survival.

**Table 3:** Association between KELIM score, NLRΔ, and ORR.

ORR	KELIM Score ≥ 1	KELIM Score < 1	NLR∆ Positive/Stable	NLR∆ Negative
Favorable response (CR + PR + SD) (*n*, %)	64 (100%)	8 (53.3%)	68 (98.5%)	4 (40%)
Unfavorable response (PD) (*n*, %)	0 (0%)	7 (46.6%)	1 (1.5%)	6 (60%)

ORR = overall response rate; CR = complete response; PR = partial response; SD = stable disease; PD = progressive disease.

## Data Availability

The data presented in this study are available on request from the corresponding author.
